# Binaral Rivalry in the Presence of Visual Perceptual and Semantic Influences

**DOI:** 10.1371/journal.pone.0047317

**Published:** 2012-10-09

**Authors:** Jennifer Chen, Wen Zhou, Denise Chen

**Affiliations:** 1 Psychology Department, Rice University, Houston, Texas, United States of America; 2 Key Laboratory of Mental Health, Institute of Psychology, Chinese Academy of Sciences, Beijing, China; 3 Department of Neurology, Baylor College of Medicine, Houston, Texas, United States of America; Duke University, United States of America

## Abstract

When two different odorants are presented simultaneously to the two nostrils, we experience alternations in olfactory percepts, a phenomenon called binaral rivalry. Little is known about the nature of such alternations. Here we investigate this issue by subjecting unstable and stable olfactory percepts to the influences of visual perceptual or semantic cues as participants engage in simultaneous samplings of either two different odorants (binaral) or a single odorant and water (mononaral), one to each nostril. We show that alternations of olfactory percepts in the binaral setting persist in the presence of visual perceptual and semantic modulations. We also show that perceptual cues have a stronger effect than semantic cues in the binaral case, whereas their effects are comparable in the mononaral setting. Our findings provide evidence that an inherent, stimulus-driven process underlies binaral rivalry despite its general susceptibility to top-down influences.

## Introduction

Multistable phenomena refer to perceptual alternations that are spontaneous, but can still be subject to bottom-up perceptual modulation and top-down voluntary control [1], [2]. The extent to which ambiguities are resolved with bottom-up and top-down influences provides important clues to the mechanism of multistable perception [3]. Ambiguous percepts that involve competition between low-level features (e.g., binocular rivalry) are subject to a greater influence of bottom-up processing such as contrast, brightness, and visual context but are fairly resistant to top-down cognitive control [1], [4]. By contrast, competitions between high-level pattern representations (e.g., Necker cube) are more readily resolved with top-down processing [4], which is facilitated by the recruitment of the dorsolateral prefrontal cortex and other frontal regions [5], [6].

In olfaction, alternations of individual smells occur when two different smells are simultaneously inhaled in each of the two nostrils in a phenomenon called binaral rivalry [7]. It remains unknown, however, whether binaral rivalry is susceptible to different levels of visual modulation, and for that matter, whether rivalry in olfaction shares similar neural mechanisms that govern rivalry in vision. Here we address this by investigating the potency of two types of visual cues, pictures and words, on modulating olfactory rivalry.

Previous studies have shown that visual perceptual and semantic information exerts strong top-down influences [9] on odor pleasantness [8]–[10], intensity [10]–[12], detection [13], [14], discrimination [14], [15], and identification [16], [17]. Color the white wine red and the wine experts mistake its smell for red [16]. “A rose by any other name would smell as sweet” to Juliet but isovaleric acid labeled as cheese smells more pleasant than the same smell labeled as body odor [8].

Although both evoke perceptual and semantic object representations [18], [19], pictures and words are distinguishable at the level of cognitive [19], [20] and neural representations [18], [21]–[23]. Pictures embody concrete perceptual attributes whereas words represent abstract symbols. Unlike words, pictures automatically engage multiple representations with perceptual properties of smell, sight, and sound [24]–[26], at a level below conscious semantic awareness [26]. Pictures evoke forward connections from early visual areas via bottom-up stimulus-dependent modulation whereas words induce explicit semantic processing by reactivating semantic representations via top-down modulation [27]–[31]. Moreover, whereas both olfactory and visual perceptions are essentially processes of object recognition [32], the inherent associations between smells and words are loose and less apparent (olfaction does not lend itself readily to verbal descriptions) [33], possibly due to neuroanatomical distance between the two systems or interference from shared cortical resources [34], [35]. All these suggest that pictures bind with olfactory percepts at an earlier stage in the olfactory pathway than words do.

At the same time, it is known from the sensory integration literature that congruent multisensory information has the greatest effect on the sensory processing in the modality whose input is most ambiguous [36]–[38]. We therefore believe that visual modulation of olfaction will be stronger when olfactory perception is in flux.

With these considerations in mind, we employ the paradigm of visual modulation of olfaction and the well-recognized distinction between pictures and words to probe the resiliency of binaral rivalry.

## Materials and Methods

### Ethics Statement

All participants gave written informed consent for participation and all procedures were conducted with the approval of Rice University's Institutional Review Board.

### Participants

90 healthy nonsmokers (45 males, 45 females, mean age = 21.11 yrs; SEM = 0.36) participated in 6 experiments, with 15 participants (comparable number of men and women) in each experiment. They reported having a normal sense of smell and no respiratory allergy or infection at the time of testing.

### Olfactory Stimuli

The olfactory stimuli consisted of purified water (8 ml) and either phenyl ethyl alcohol (PEA, a rose like smell) or n-butanol (a marker pen like smell) (each 0.5% v/v in propylene glycol, 8 ml) in the mononaral condition (rose/water and marker/water, respectively), and both PEA and n-butanol in the binaral condition (rose/marker). They were presented in identical 280 mL glass bottles, each fitted with a custom-made Teflon nosepiece. The PEA and n-butanol, rated as equally familiar (*p* = .82) to the subjects, differ in molecular structure. Compared to the aliphatic compound n-butanol, PEA, which is aromatic, was perceived as more rose-like (*p*<.001), less marker pen-like (*p*<.001), more pleasant (*p*<.001), and slightly less intense (*p* = .04). All subjects correctly matched the two odorants to the labels of ‘rose’ and ‘marker’ in the absence of visual cues prior to the actual experiment.

### Pictorial and Semantic Cues

**Figure 1:**
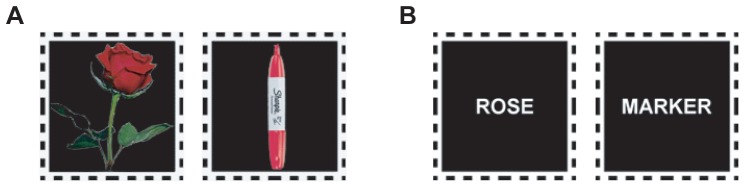
Visual stimuli. Visual stimuli consisted of pictures (A) and words (B).

The stimuli consisted of pictures (an image of a rose and an image of a marker pen, with a visual angle of about 18.18°×15.66° and 2.86°×15.66°, respectively; Figure 1A) and words (‘rose’ or ‘marker’; with a visual angle of about 5.72°×1.43° and 7.87°×1.43°, respectively; Figure 1B).

### Procedure

**Figure 2:**
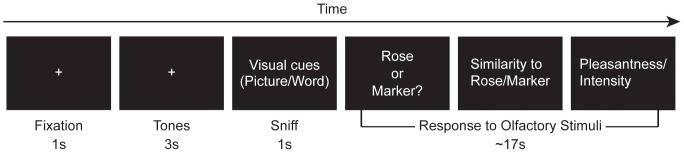
Illustration of an experimental trial. Each trial began with a fixation (1 s) followed by 2 low tones and 1 high tone (1 s each) that prepared and prompted the subjects to take a single sniff of a pair of bottles at the end of the high tone for the duration (1 s) of a visual stimulus (picture/word) while maintaining fixation on the visual stimulus. Subjects subsequently indicated whether they smelled predominantly “rose” or “marker,” and rated its similarity to the rose and marker smells, as well as its intensity and pleasantness on separate VASs with ‘not at all’ on one end of the scale and ‘extremely’ on the other end.

All olfactory samplings were made in the presence of either a picture or a word. Subjects pressed one of two buttons to indicate if they detected predominantly the rose or marker smell and then rated on a 100 unit visual analog scale (VAS) how similar (from “not at all” to “extremely”) the perceived smell was to that of rose and marker pen, respectively, and how pleasant and intense each smell was (see Figure 2 for an illustration).

Each subject completed 40 intermittent trials presented in 4 blocks to prevent adaptation [7]. A between-subject design was adopted such that each olfactory combination was paired with only one type of cues (pictures or words) in an experiment. This was necessary to minimize task-switching [39] and interference between visually presented pictures and words [21]. The side of the nostril to which a smell was presented was counterbalanced across subjects. The experimenters and the subjects were blind to the purpose of the study, the nature of the olfactory stimuli, and the side of the nostril a smell was presented to.

### Analyses

The modulatory effects of visual and semantic cues under different olfactory conditions were first explored with a repeated measures ANOVA, using the proportion of button responses for detecting predominantly a rose smell (calculated as the number of rose responses divided by the total number of trials, which equals 1 minus the proportion of button responses for detecting predominantly a marker smell) as the dependent variable, cue content (rose cues vs. marker cues) as the within-subjects factor, and cue type (pictures vs. words) and olfactory condition (rose/water vs. marker/water vs. rose/marker) as the between-subjects factors. They were further quantified by careful analyses of the similarity ratings.

The ratings on the two similarity scales (similarity to rose smell vs. similarity to marker smell) were significantly anti-correlated, *r* = −.69, *p*<.001. We thus took their difference (similarity to rose smell minus similarity to marker smell) and combined them into a single bipolar scale that ranged from −100 (extremely similar to marker smell) to 100 (extremely similar to rose smell), where 0 marked a mixed percept (50% like rose smell and 50% like marker smell) or a percept similar to neither rose nor marker smell. The difference of the scores on the combined scale in the presence of a rose cue vs. a marker cue denotes the magnitude of modulation and was then used as the dependent variable in an univariate ANOVA to assess the modulatory effects of visual perceptual and semantic cues under different olfactory conditions, in which cue type (pictures vs. words) and olfactory condition (rose/water vs. marker/water vs. rose/marker) served as the factors.

Follow-up independent sample T tests were performed where appropriate. Multiple comparisons were corrected with Bonferroni method.

To examine olfactory adaptation, we performed a repeated-measures ANOVA with intensity ratings as the dependent variable, number of samplings (1 to 40) and cue content (rose vs. marker) as the within-subjects factors, and cue type (pictures vs. words) and olfactory condition (rose/water, marker/water, rose/marker) as the between-subjects factors. Since PEA and butanol differed significantly in valence, we also assessed the relationships between perceived smell quality (reflected in smell similarity ratings) and valence (pleasantness ratings) under the mononaral and binaral settings, respectively, with bivariate Pearson correlation.

We performed Hartigan's dip test [40] to characterize the distributions of olfactory percepts under the influences of visual perceptual (pictures) versus semantic (words) cues in the binaral and mononaral conditions.

## Results

**Figure 3:**
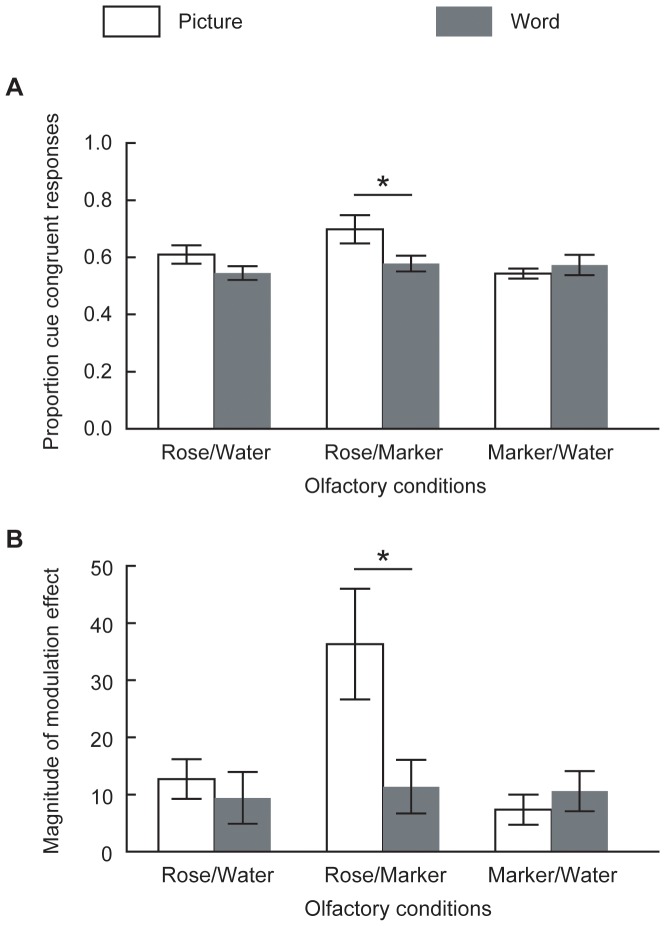
Visual perceptual versus semantic modulations of mononaral and binaral olfactory perceptions. Comparison between visual perceptual and semantic modulations of mononaral (rose/water, marker/water) and binaral (rose/marker) olfactory perception as reflected in (A) proportion of cue-congruent button responses reporting detecting the cue-congruent smell, and (B) magnitude of cue-induced perceptual alternations on the combined bipolar similarity scale. Compared with words, pictures significantly increased cue-congruent button responses and biased similarity ratings to the cue-congruent end under binaral but not mononaral settings. The proportion of cue-congruent olfactory responses was calculated as the number of cue-congruent button responses (e.g. smelling a rose smell in the presence of a rose picture or the word ‘rose’) divided by the total number of trials. The magnitude of modulation effect was calculated as the distance between the similarity scores on the combined scale in the presence of a rose cue vs. a marker cue. Error bars represent standard errors of the mean. *p<.05.

We first examined the proportion of cue-congruent button press responses in a repeated measures ANOVA with cue content (rose cues vs. marker cues) as the within-subjects factor, and cue type (pictures vs. words) and olfactory condition (rose/water vs. marker/water vs. rose/marker) as the between-subjects factors. We showed that overall, subjects were inclined to detect the cue-congruent smell under both mononaral and binaral settings, *F*(1, 84) = 46.89, *p*<.001, but more so in the latter case, as indicated by a significant interaction between cue content (rose cues vs. marker cues) and olfactory condition (rose/water vs. marker/water vs. rose/marker), *F*(2,84) = 3.27, *p* = .04, as well as a significant difference between the binaral and mononaral (rose/water and marker/water combined) conditions in the follow-up t test, *t*(87) = 2.40, *p* = .02. Under the mononaral settings where the olfactory inputs were unambiguous, the average odor identification accuracies for the rose and the marker smells were 76% and 86%, respectively. There was also a significant interaction between cue type (pictures vs. words) and olfactory condition (rose/water vs. marker/water vs. rose/marker), *F*(2,84) = 3.39, *p* = .04. Figure 3A shows that pictures led to significantly greater modulation than words in the binaral condition (*p* = .03) but not in the mononaral conditions (*ps* = .15 and .89 for rose/water and marker/water, respectively). In other words, visual perceptual cues significantly outweighed semantic cues when the olfactory input was equivocal rather than unequivocal.

The above were further characterized by alternations in the smell similarity ratings. There was a significant main effect of olfactory condition, *F*(2,84) = 4.63, *p* = .01, in which vision exerted a larger influence on olfaction in the binaral than the mononaral conditions, *t*(87) = 2.88, *p* = .01. There was also a significant cue type (pictures vs. words) by olfactory condition (rose/water vs. marker/water vs. rose/marker) interaction, *F*(2,84) = 3.88, *p* = .02, contributed by the larger modulation of pictures in the binaral condition (*p* = .001, Figure 3B). Similar effects were not exhibited in either mononaral condition (*ps* = .66 and .67 for rose/water and marker/water, respectively). The means of maximum similarity to rose rating were comparable in the mononaral and binaral conditions (*p* = .12), as were the mean maximum similarity to marker ratings (*p* = .98).

**Figure 4:**
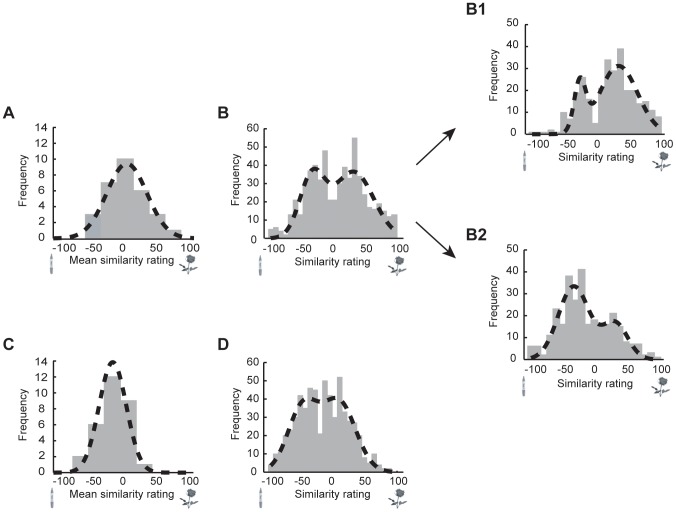
Histograms of visual perceptual versus semantic modulations of binaral similarity ratings. How biased a subject was towards smelling rose or marker, as indicated by their mean similarity ratings, follows a normal distribution in the presence of both pictures (A) and words (C). Their individual similarity ratings across all trials, however, form a bimodal distribution in both cases, with a larger separation between the two peaks (local maxima) in the presence of pictures (B) as compared to words (D). Critically, binaral rivalry retains its own dynamics even under the strong influences from the pictorial cues of rose (B1) and marker (B2). In Figure B, Figure B1, Figure B2, and Figure D, the distributions are modeled with the sum of two normal distributions (dotted curve):
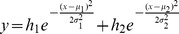
_,_ where 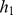, 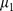, 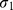 are the height, mean, and standard deviation, respectively, of the first normal distribution, and 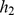 , 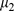, 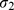 are the height, mean, and standard deviation, respectively, of the second normal distribution.

The magnitude of visual modulation in the binaral setting can be readily visualized with the histogram of the bipolar similarity ratings across all the subjects and trials in the picture and word conditions. As illustrated in Figure 4, how biased a subject was towards smelling ‘rose’ or ‘marker’, as reflected by his/her mean similarity rating, followed a normal distribution in both the binaral picture (Figure 4A) and word (Figure 4C) conditions, but across all trials in each of these two conditions (600 ratings from 15 subjects, each with 40 samplings), similarity ratings formed a bimodal distribution (Figure 4B & 4D). The bimodal separation is more distinct in the picture condition than the word condition, showing a greater susceptibility to the visual modulation (Hartigan's dip test, *p*<.001 for pictures and *p*<.05 for words). A blow up of the bimodal picture condition shows that the distribution is skewed in the direction of the congruent picture (Figure 4B1 & 4B2). The corresponding dip tests in the mononaral conditions were statistically insignificant (*ps*>.23).

There was a significant linear reduction in perceived smell intensity over time, *F*(1,89) = 30.73, *p*<.001, but it was not correlated with smell quality under the binaral settings, *rs* = .22 and .22, *ps* = .23 and .25 for rose and marker cues, respectively. Instead, pleasantness ratings directly mirrored smell similarity ratings in both the binaral settings, *rs* = .81 and .46, for rose and marker cues, respectively, *ps*<.01, and the mononaral settings, *rs* = .62 and .66, for rose and marker cues, respectively, *ps*<.001. Irrespective of how strong the perceived smell was, subjects tended to rate it as more pleasant when it smelled more like rose, and vice versa.

Critically, olfactory alternations persist in the binaral condition (Figure 4B1 & 4B2) despite greater susceptibility to visual modulation, suggesting that the rivalry has its own intrinsic dynamics that are resistant to modulations.

## Discussion

The tendency to detect the cue-congruent smell more under the binaral than the mononaral settings is consistent with sensory integration understanding that ambiguous information in one sense is subject to the influence of less ambiguous information in another sense [36], [37].

Neuroanatomically, pathways linking retinal and olfactory inputs have been reported in primates and other mammals, involving such structures as the piriform cortex, olfactory tubercle, cortical region of the medial amygdala, lateral hypothalamus, and the bed nucleus of the stria terminalis [41]–[43]. Recent neurophysiological and neuroimaging studies have associated the processing of olfactory and visual information with activities in the piriform cortex, insular, hippocampus, and in particular the orbitofrontal cortex [8], [13], [44], [45].

Our finding that binaral rivalry is more readily modulated by visual perceptual cues than by semantic information, along with the existing evidence that pictures and smells have greater binding propensity than do words and smells [19]–[35], suggests that binaral rivalry involves an early stage of olfactory hierarchy. Quite possibly this occurs at the perceptual object representation level, which is not directly accessible to semantic representations. Moreover, while binaral rivalry is susceptible to visual perceptual and semantic influences, it carries its own dynamics.

In conclusion, olfaction has long been considered a secondary sense in humans that is dominated by vision, and easily swayed by top-down influences. Here we probe the effects of visual perceptual versus semantic influence on olfaction when the olfactory percepts are in flux. We show that binaral olfaction is subject to even greater visual influence than mononaral olfaction. Interestingly, we show that binaral olfaction, despite its greater proneness to visual influence, does not succumb to vision. Furthermore, and specific to the binaral conditions, we show that perceptual information exerts a greater bias of olfactory perception than semantic information. Together, our findings point to the greater role of an inherent, automatic stimulus-driven process in binaral rivalry. Such a process is similar to that implicated in binocular rivalry, but has been little studied in olfaction.
